# NLRP3 inflammasome activation contributes to development of alopecia areata in C3H/HeJ mice

**DOI:** 10.1111/exd.14432

**Published:** 2021-07-26

**Authors:** Kei Hashimoto, Yoshihito Yamada, Kota Sekiguchi, Sachi Mori, Tatsumi Matsumoto

**Affiliations:** ^1^ Kyoto R&D Center Maruho Co., Ltd. Kyoto Japan

**Keywords:** alopecia areata, MCC950, NLRP3 inflammasome

## Abstract

Alopecia areata (AA) is an autoimmune non‐scarring hair loss disease. Recently, several reports have suggested that innate immune systems such as interferon‐α (IFN‐α)‐producing plasmacytoid dendritic cells and NOD‐like receptor family pyrin domain‐containing protein 3 (NLRP3) inflammasomes play a role in the pathogenesis of AA. However, critical studies about their involvement in the initiation of AA have not yet been reported. Therefore, we investigated the expression of innate immune cytokines in serum and skin, and examined the effect of a selective NLRP3 inhibitor, MCC950, on AA in C3H/HeJ mice, induced by transferring cultured skin‐draining lymph node cells. IFN‐α production was upregulated in lesions of AA‐affected mice, and interleukin‐1β in serum and skin was highly expressed before onset as well as postonset. Furthermore, MCC950 treatment prevented AA development and promoted hair growth in AA mouse models by reducing NLRP3 signalling and Th1/Tc1 chemokines and cytokines in the skin. These results suggest that NLRP3 inflammasome contributes to AA onset and chronicity, and NLRP3 inhibitor may be a potential therapeutic agent for AA.

## 1 INTRODUCTION

Alopecia areata (AA) is an autoimmune disease characterized by non‐scarring hair loss. Although AA is not life‐threatening, it has a detrimental impact on quality of life. It has been suggested that AA develops in anagen hair follicles (HF) through collapse of constitutive immune privilege (IP). This hypothesis is supported by infiltration of lymphocytes, natural killer (NK) cells, Langerhans cells, dendritic cells (DCs) and macrophages into the peribulbar area of anagen HFs. It has also been demonstrated that MHC class I and II expression increases and IP guardians decrease in AA lesions.^1^, ^2^, ^3^

What triggers IP collapse is still under debate. Several studies have indicated that the innate immune system plays an important role in the pathogenesis of AA, based on clinical reports concerning associations with viral infection,^4^, ^5^, ^6^ psychological stress^7^ and oxidative stress.^8^ In innate immunity, pattern recognition receptors (PRRs), such as toll‐like receptors (TLRs) and NOD‐like receptor family pyrin domain‐containing protein 3 (NLRP3), are key regulators. Activation of PRR signalling is essential for host defense against infection, whereas overactivation of PRRs often causes uncontrolled inflammation and autoimmune diseases.^9^, ^10^

TLRs have been identified from TLR1 to TLR11 in humans. Several TLRs are localized in the endosome to detect nucleic acids derived from bacteria, viruses and damaged cells. Endogenous nucleic acids, which are not recognized by the endosomal TLRs under homeostatic conditions, activate the TLRs and trigger development of autoimmune diseases^9^ such as psoriasis,^11^ systemic lupus erythematosus (SLE),^12^ rheumatoid arthritis (RA),^13^ diabetes mellitus^14^ and systemic sclerosis (SSc).^15^ In AA patients, expressions of TLRs 3, 7, 8 and 9 are reported to be upregulated in peripheral blood mononuclear cells^16^, ^17^ and/or around lesional HFs.^17^ Stimulation of TLRs 7 and 9 mediates interferon (IFN)‐α secretion from plasmacytoid DCs (pDCs). pDCs are present in the peribulbar area of AA patients,^18^ and type 1 interferon‐inducible mexovirun protein A is expressed in AA lesions.^19^ A recent study showed that IFN‐α‐producing pDCs were infiltrated around HFs not only in AA lesions but also in non‐lesional areas of AA‐affected C3H/HeJ mice, and that intradermal injection of pDCs induced AA lesions in normal C3H/HeJ mice.^20^ IFN‐α‐producing pDCs are known to play a role in scarring alopecia such as Lupus erythematosus‐associated alopecia.^21^

NLRP3 is activated via the P2X7 receptor, assembling with apoptosis‐associated speck‐like protein containing a caspase recruitment domain (ASC), and pro‐caspase‐1 into inflammasomes. The NLRP3 inflammasome promotes secretion of interleukin (IL)‐1β and IL‐18. Several studies have revealed their significance in autoimmune disorders such as SLE, RA and SSc.^10^ In AA patients, NLRP3 inflammasome components and IL‐1β are reported to be highly expressed in the outer root sheath of HF.^22^ Serum levels of IL‐18 in patients with extensive AA are significantly higher than in healthy controls,^23^ and single‐nucleotide polymorphisms in *IL*‐*18* (re187238 and rs549908) are related to the susceptibility to AA in Koreans.^24^

Thus, several studies have suggested a contribution of IFN‐α‐producing pDCs and NLRP3 inflammasome activation to AA pathogenesis. However, their roles in the initiation of AA are yet to be clarified. To define their roles in the onset of AA, we investigated the expression of IFN‐α and NLRP3‐related factors, comparing before and after AA onset, using an AA mouse model reflecting AA pathogenesis.^25^ Furthermore, we studied the therapeutic effect of an NLRP3 inhibitor on AA. This is the first report to demonstrate that inhibition of NLRP3 inflammasome may be a promising therapeutic approach to AA.

## 2 METHODS

### 2.1 Mice and induction of AA

Female 7‐week‐old C3H/HeJ mice were obtained from Japan SLC (Shizuoka, Japan). All mice were housed in specific pathogen‐free conditions. They received food pellets for long‐term breeding (CR‐LPF; Oriental Yeast) and ultraviolet sterile water *ad libitum*. All animal experimental procedures were approved by the Ethics Committee for Animal Experiments of Maruho (Kyoto, Japan) and conducted in accordance with the Guiding Principles for the Care and Use of Laboratory Animals at Maruho.

The AA mouse model was induced by transferring *in vitro* expanded lymph node (LN) cells from AA‐affected mice, as previously described,^26^, ^27^ with minor modifications. Briefly, skin‐draining LN cells (cervical, axillary, inguinal and popliteal LN) were obtained from previously AA‐affected mice (induced by transferring expanded LN cells) and prepared in RPMI 1640 medium (Thermo Fisher Scientific) supplemented with 10% FBS (MP Biomedicals), 100 U/ml Penicillin‐Streptomycin (Nacalai Tesque), 2 mM GlutaMAX (Thermo Fisher Scientific), 30 U/ml recombinant human IL‐2 (Roche Diagnostics), 25 ng/ml recombinant mouse IL‐7 (R&D Systems) and 50 ng/ml recombinant mouse IL‐15 (R&D Systems). Cells were activated with Dynabeads Mouse T‐Activator CD3/CD28 (Thermo Fisher Scientific) and cultured for 7 days. Naive 8‐week‐old C3H/HeJ mice were injected intradermally with 2 × 10^7^ cells/mouse.

Mice were divided into the following four groups (*N* = 4 per group): naive mice (without transferred LN cells); preonset mice (with transferred LN cells but without hair loss); AA mice at initial phase (with hair loss localized in less than half of the abdominal area for less than 2 weeks); and AA mice at severe phase (with hair loss in whole abdominal area and less than half of dorsal area for more than 4 weeks).

### 2.2 MCC950 treatment

Among AA‐affected mice at initial phase (hair growth index between 50 and 100), mice were randomized into two groups: MCC950‐treated or PBS‐treated mice (*N* = 5 per group). Mice were injected subcutaneously with MCC950 (20 mg/kg) (Selleck Chemicals) or PBS (Sigma‐Aldrich) on days 0, 1, 3, 6, 8, 10, 13, 15, 17, 20, 22, 24, 27, 29, 31, 34, 36, 38 and 41. Hair growth was evaluated every week after the first administration. For analysis of pathological characteristics, mRNA expressions in skin and cytokine productions in serum, AA‐affected mice were injected subcutaneously with MCC950 or PBS on days 0, 1, 3 and 6 (*N* = 3 per group).

### 2.3 Evaluation of hair growth

Hair growth was assessed in the square area (2 × 3 cm) on abdominal skin. Hair growth was evaluated using the 4‐point score with definition as follows: 0 (no hair); 1 (non‐dense short hairs); 2 (non‐dense intermediate length hairs); and 3 (normal length and density hairs). The percentage of the area with each score point in 2 × 3 cm area was measured using ImageJ software (National Institutes of Health). Hair growth index was calculated as the sum of multiplying each hair growth score by the percentage of scored area.

### 2.4 Real‐time polymerase chain reaction analysis

To determine mRNA expressions, total RNA was isolated from homogenized skin samples (lesions, hair‐loss areas; non‐lesions, normal hairy areas remote from lesions) using RNeasy Fibrous Tissue Mini Kit (Qiagen). cDNA was synthesized by reverse transcription of total RNA using High Capacity cDNA Reverse Transcription Kit (Thermo Fisher Scientific). Amplification was performed using TaqMan^®^ Gene Expression Master Mix and TaqMan Gene Expression Assay (Applied Biosystems^™^). The primer sets purchased from Thermo Fisher Scientific were as follows: *Ager* (Mm01134790_g1), *Casp1* (Mm00438023_m1), *Cxcl9* (Mm00434946_m1), *Cxcl10* (Mm00445235_m1), *Cxcl11* (Mm00444662_m1), *Gzmb* (Mm00442837_m1), *Hspa1a* (Mm01159846_s1), *Hmgb1* (Mm00849805_gH), *Ifng* (Mm01168134_m1), *Irak4* (Mm00459443_m1), *Irf7* (Mm00516793_g1), *Nlrp3* (Mm00840904_m1), *Pycard* (Mm00445747_g1), *Traf3* (Mm00495752_m1) and *Gapdh* (Mm99999915_g1). The results were normalized to GAPDH and analysed with QuantStudio^™^ 7 Flex and StepOnePlus^™^ real‐time polymerase chain reaction (PCR) systems (Thermo Fisher Scientific).

To determine microRNA expressions, total RNA was extracted from whole blood using miRVana^™^ PARIS kit (Thermo Fisher Scientific). cDNA was synthesized using TaqMan^™^ Advanced miRNA cDNA Synthesis Kit. Amplification was performed using TaqMan Fast Advanced Master Mix and TaqMan Advanced miRNA Assay. The primer sets purchased from Thermo Fisher Scientific were as follows: mmu‐miR‐30b‐3p, mmu481756_mir; mmu‐miR‐150‐5p, mmu480947_mir; mmu‐miR‐423‐3p, and mmu478327_mir. The results were normalized to mmu‐miR‐423‐3p and analysed with the StepOnePlus System.

### 2.5 Enzyme‐linked immunosorbent assay and multiplex

Skin samples were homogenized in PBS containing 0.1% Tween 20 (Nacalai Tesque) and 1% Protease Inhibitor Cocktail Set III (Merck) and centrifuged. Blood samples were collected under anaesthesia, and serum was isolated after coagulation and centrifugation. The concentrations of IFN‐α, heat shock protein 70 (HSP70) and IL‐18 were measured by enzyme‐linked immunosorbent assay (ELISA) using VeriKine‐HS Mouse IFN‐α All Subtype ELISA Kit (PBL Assay Science), Mouse HSP70 ELISA kit (CUSABIO) and Mouse IL‐18 SimpleStep ELISA kit (Abcam). The concentrations of IL‐1β, IFN‐γ, tumor necrosis factor (TNF)‐α and IL‐17A were measured using Bio‐Plex Multiplex Immunoassay System (Bio‐Rad) with Bio‐Plex Pro Mouse Cytokine IL‐1β Set and Bio‐Plex Pro Mouse Cytokine Th1/Th2 Assay (Bio‐Rad).

### 2.6 Histological analysis

Skin samples from MCC950‐ and PBS‐treated mice were collected and fixed in 10% formalin. Paraffin‐embedded sections (3 μm) were stained with haematoxylin and eosin (HE). The stained slides were subsequently evaluated using the SLIDEVIEW VS200 digital slide scanner (Olympus).

### 2.7 Statistical analysis

Data are expressed as mean ± standard error of the mean (SE). Statistical analysis was conducted using Student's *t* test or Dunnett's multiple comparison test, implemented using EXSUS software (CAC Croit). A *p*‐value less than 0.05 was considered statistically significant.

## 3 RESULTS

### 3.1 IFN‐α signal pathway is activated in lesion at the initial phase

**FIGURE 1:**
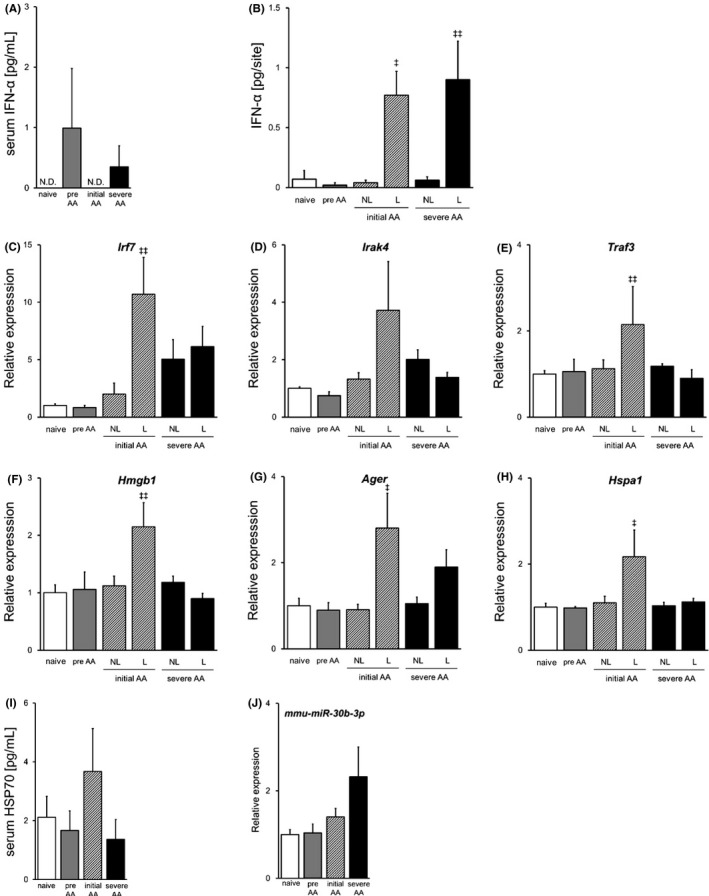
IFN‐α signal pathway is activated in lesions in the initial phase of alopecia areata. IFN‐α levels in serum (A) and skin (B) from naive, preonset, initial AA and severe AA mice were measured by ELISA. mRNA expressions of IRF7 (C), IRAK4 (D), TRAF3 (E), HMGB1 (F), RAGE (G) and HSP70 (H) were assessed by RT‐PCR. (I) Serum HSP70 level was measured by ELISA. (J) Expression of *mmu*‐*miR*‐*30b*‐*3p* in blood was assessed using RT‐PCR. Skin samples were collected from non‐lesional areas (NL; normal hairy area) and lesions (L; hair loss area). Data are expressed as mean ± SE (*N* = 4). ^‡^, *p* < 0.05. ^‡‡^, *p* < 0.01. vs. naive (Dunnett's multiple comparison test). Abbreviations: AA, alopecia areata; ELISA, enzyme‐linked immunosorbent assay; RT‐PCR, reverse transcription polymerase chain reaction; SE, standard error; and N.D., not detected

pDCs and NLRP3 inflammasome have been suggested to be initiation factors in AA. We first examined pDC activation by comparing the disease phases. The IFN‐α level in serum and skin of AA mice with different disease phases was assessed using ELISA (Figure 1A, B). IFN‐α concentration in serum was detectable only in preonset and severe AA mice. Whereas IFN‐α levels in non‐lesional areas of AA mice were as low as those of naive and preonset mice, IFN‐α levels in lesions were increased compared with non‐lesional areas. We also examined the expression of factors that are crucial for IFN‐α production (Figure 1C–E). mRNA expression of IRF7 (interferon regulatory factor 7) was higher in lesions of initial AA mice and non‐lesional areas and lesions of severe AA mice. IRAK4 (interleukin‐1 receptor‐associated kinase 4) and TRAF3 (tumor necrosis factor receptor‐associated factor 3) expressions in lesion of initial phase AA mice were increased, and mRNA expression of IRAK4 in non‐lesional areas was higher than in lesions of severe AA mice. These results demonstrate that the IFN‐α signal pathway is locally activated in lesional areas at the initial phase.

IFN‐α production via TLR7/9 in pDCs is activated via several signal pathways suggested to play an important role in several autoimmune diseases. A complex of HMGB1 (high mobility group box‐1) and DNA binds to RAGE (receptor for advanced glycation end‐products) and induces IFN‐α production.^28^ HSP70 potentiates DNA‐induced IFN‐α production.^29^ To elucidate the trigger of pDC activation and IFN‐α production, we determined mRNA expressions of HMGB1, RAGE and HSP70 (Figure 1F–H). The expressions of HMGB1, RAGE and HSP70 increased in lesions of initial phase AA mice, which was similar to IFN‐α. Serum level of HSP70 was increased in initial phase AA mice (Figure 1I), and the expression of *mmu*‐*miR*‐*30b*‐*3p*, which is reported to inhibit HSP70 expression, was higher in blood of AA mice, and particularly elevated in severe AA mice (Figure 1 J). Taken together, HMGB1/RAGE and HSP70 signal pathways were activated and IFN‐α production was increased in lesions at the initial phase.

### 3.2 NLRP3 Inflammasome signal pathway contributes to development and exacerbation of AA

**FIGURE 2:**
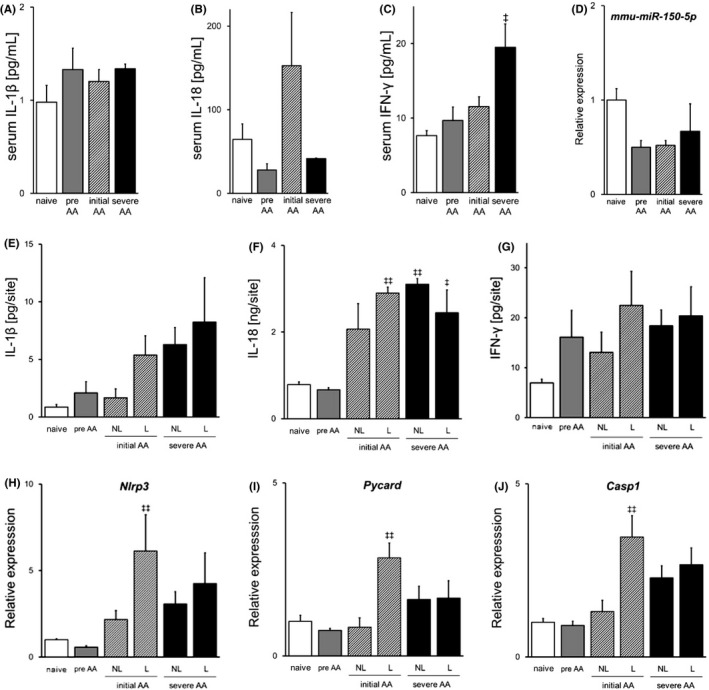
Inflammasome signal pathway‐related factors are increased in both initial and severe phases of alopecia areata. (A‐C, E‐G) IL‐1β, IL‐18 and IFN‐γ levels in serum and skin from naive, preonset, initial AA and severe AA mice were measured by multiplex. (D) Expression of *mmu*‐*miR*‐*150*‐*5p* in blood was assessed by RT‐PCR. (H‐J) mRNA expressions of NLRP3, ASC and caspase‐1 in the skin were assessed by RT‐PCR. Data are expressed as mean ± SE (*N* = 4). ^‡^, *p* < 0.05. ^‡‡^, *p* < 0.01. vs. naive (Dunnett's multiple comparison test). Abbreviations: AA, alopecia areata; RT‐PCR, reverse transcription polymerase chain reaction; and SE, standard error

Next, to investigate whether NLRP3 inflammasome contributes to AA development, we evaluated the production of IL‐1β and IL‐18 in serum and skin by comparing naive, preonset, initial AA and severe AA mice. Serum IL‐1β level was subtly higher in preonset and AA mice than in naive mice (Figure 2A). Serum IL‐18 level was increased in initial AA mice and decreased in preonset and severe AA mice (Figure 2B). Serum level of IFN‐γ, that is reported to play a crucial role in the pathogenesis of AA, was significantly increased in severe AA mice (Figure 2C). The blood level of *mmu*‐*miR*‐*150*‐*5p*, which is reported to inhibit IL‐18 expression, was lower in preonset, initial AA and severe AA mice than in naive mice (Figure 2D). IL‐1β production in skin was increased in preonset and AA mice, and the level was elevated depending on the progression of AA (Figure 2E). IL‐18 production was increased in non‐lesional areas and lesions of AA mice, and the level was similar between initial AA and severe AA (Figure 2F). Comparing IL‐1β and IL‐18 production, IL‐18 level was significantly higher than IL‐1β level both in serum and in skin. Furthermore, IL‐1β production, but not IL‐18 production, was increased in preonset mice. These data demonstrate that IL‐1β production increased before onset of AA, and IL‐1β and IL‐18 production increased in non‐lesional areas and in lesions after onset. IFN‐γ production in skin tended to increase in mice injected with lymph node cells compared to naïve mice (Figure 2G). mRNA expressions of NLRP3 were increased both in non‐lesional areas and in lesions of initial and severe AA mice, particularly in lesions of initial AA mice (Figure 2H). mRNA expressions of NLRP3 inflammasome components, ASC (*Pycard*) and caspase‐1, were increased in lesions of initial AA mice and non‐lesional areas and lesions of severe AA mice, and the level was similar between non‐lesional areas and lesions at the severe phase (Figure 2,I J). These data demonstrate that NLRP3 inflammasome signalling in skin was activated at the initial phase and remained activated at the severe phase, suggesting that NLRP3 inflammasome contributes to aggravation as well as onset of AA.

### 3.3 NLRP3 inflammasome inhibitor MCC950 ameliorates AA in the mouse model

**FIGURE 3:**
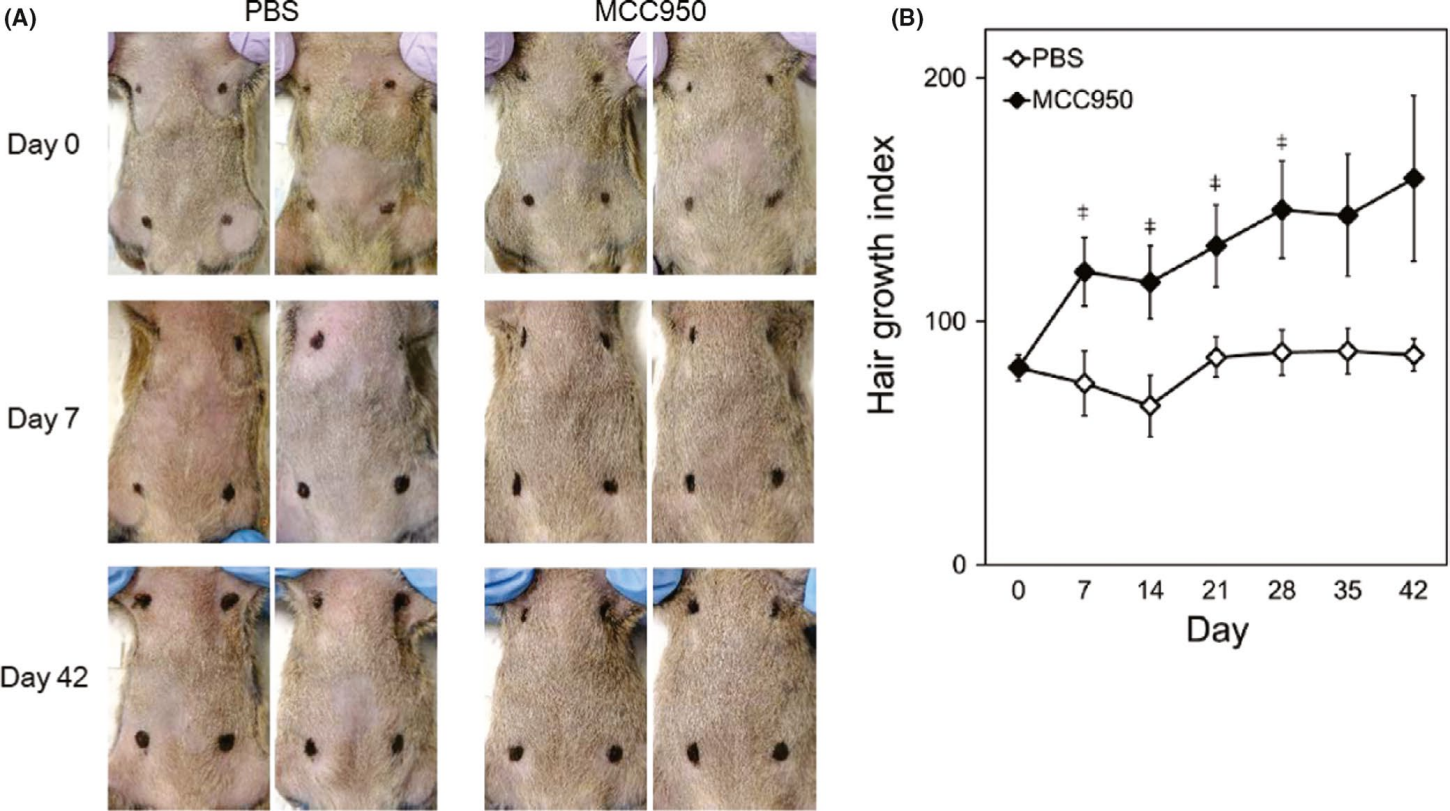
Effect of MCC950 on hair growth in alopecia areata mouse model. AA mice were subcutaneously injected with MCC950 (20 mg/kg) or PBS on days 0, 1, 3, 6, 8, 10, 13, 15, 17, 20, 22, 24, 27, 29, 31, 34, 36, 38 and 41. (A) Representative abdominal skin of AA mice before (day 0) and after administration (days 7 and 42). (B) Hair growth was evaluated every week. Data are expressed as mean ± SE (*N* = 5). ^‡^, *p *< 0.05. (Student's *t* test). Abbreviations: AA, alopecia areata; RT‐PCR, reverse transcription polymerase chain reaction; and SE, standard error

Finally, we investigated the therapeutic effect of a selective NLRP3 inflammasome inhibitor, MCC950, on AA in the mouse model. MCC950 or PBS was subcutaneously injected into AA mice for 6 weeks. In MCC950‐treated mice, hair growth was observed from 7 days up to 42 days after the first administration (Figure 3A, B). Hair growth index increased from 81.1 ± 2.4 to 158.8 ± 34.0 in MCC950‐treated mice, whereas it changed from 80.9 ± 5.4 to 86.4 ± 6.6 in PBS‐treated mice.

**FIGURE 4:**
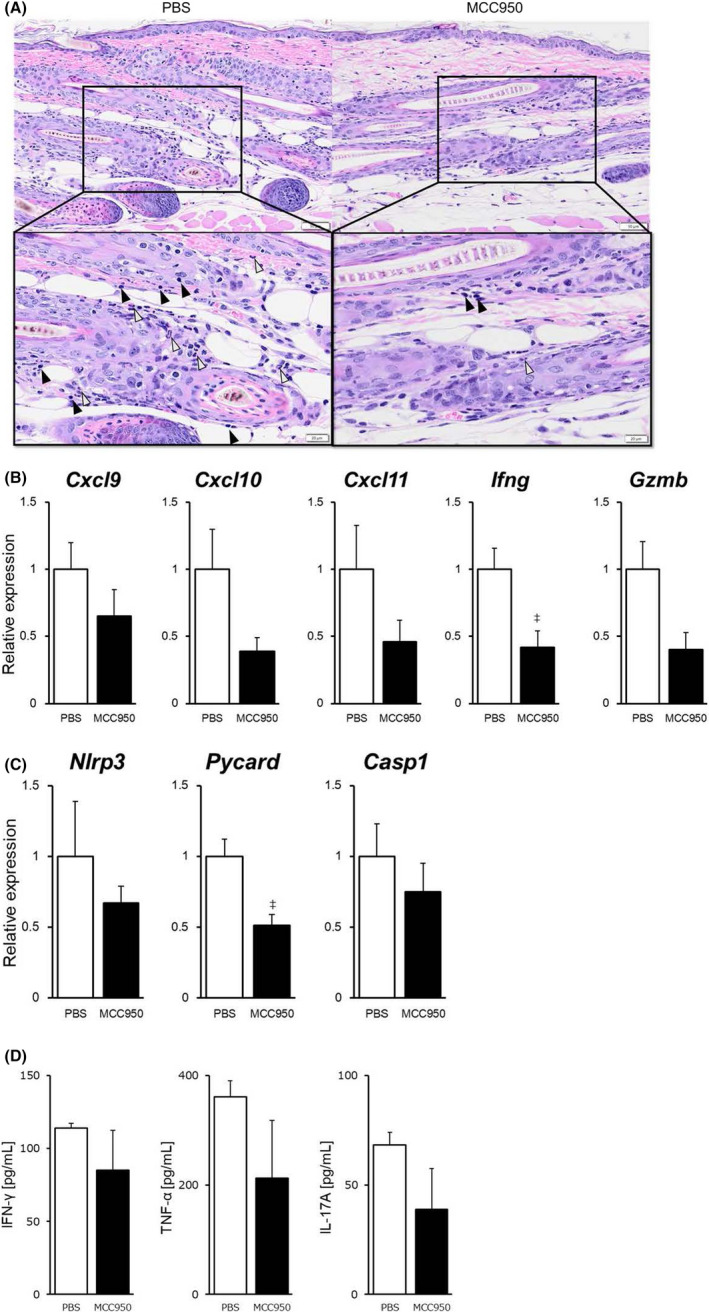
Effect of MCC950 on immune pathophysiological changes in alopecia areata mouse model. AA mice were subcutaneously injected with MCC950 (20 mg/kg) or PBS on days 0, 1, 3 and 6. (A) HE staining of the skin from PBS‐ and MCC950‐treated mice on day 7. Neutrophils and lymphocytes are marked with white and black arrows, respectively. (B, C) mRNA expressions of CXCL9/10/11, IFN‐γ, granzyme B, NLRP3, ASC and caspase‐1 in skin were assessed by RT‐PCR, and the expression levels are shown relative to PBS‐treated mice. (D) IFN‐γ, TNF‐α and IL‐17A levels in serum were measured by multiplex. Data are expressed as mean ± SE (*N* = 3). ^‡^, *p* < 0.05. (Student's *t* test). Abbreviations: AA, alopecia areata; RT‐PCR, reverse transcription polymerase chain reaction; and SE, standard error

As shown in Figure 3B, the increase in hair growth index was observed even 7 days after the first MCC950 administration and was maintained up to 42 days. Therefore, to elucidate the effect of MCC950 on cytokines, chemokines and NLRP3 inflammasome signalling in AA mice, we investigated pathological characteristics, mRNA expressions in the skin and cytokine production in serum on day 7. HE staining revealed that MCC950 treatment decreased infiltration of inflammatory cells such as neutrophils and lymphocytes (Figure 4A). MCC950 treatment decreased mRNA expression of Th1/Tc1 chemokines (CXCL9/10/11), Th1/Tc1 cytokine (IFN‐γ) and cytotoxic marker (granzyme B) in the skin (Figure 4B). mRNA expressions of NLRP3 inflammasome‐related factors (NLRP3, ASC and caspase‐1) were reduced by MCC950 treatment (Figure 4C). Serum levels of cytokines (IFN‐γ, TNF‐α and IL‐17A) were lower in MCC950‐treated mice than in PBS‐treated mice (Figure 4D).

## 4 DISCUSSION

AA is thought to arise as a consequence of the collapse of local IP with cytotoxicity induced by CD8^+^ T cells and NK cells.^1^ Although several studies imply that AA results primarily from dysregulation of innate immunity, similar to other autoimmune diseases,^3^ the trigger of IP collapse has not been clarified. Here, we compared serum and skin levels of innate immune‐related factors between naive mice, preonset mice and AA‐affected mice (initial phase and severe phase). This is the first study to demonstrate that NLRP3 inflammasome activation contributes to development and aggravation of AA and that NLRP3 inhibition may have potential for AA therapy.

In this study, we focused on IFN‐α‐producing pDCs and NLRP3 inflammasome in innate immunity, and investigated their contributions to AA onset using a C3H/HeJ AA mouse model, which is the most popular and well‐defined model for AA. First, we found that IFN‐α in serum was upregulated in preonset mice, whereas its expression in skin was unchanged (Figure 1A, B). Skin IFN‐α level was upregulated in lesions of initial AA and severe AA mice (Figure 1B). Our data demonstrate that IFN‐α (all subtypes) protein level was higher in lesions than in non‐lesional areas, whereas a previous report showed that IFN‐α2 and IFN‐α4 mRNA expressions were higher in non‐lesional areas than in lesions.^20^ This discrepancy could be caused by differences of IFN‐α subtype, definition of non‐lesions (normal hairy areas remote from lesions in our study; the vicinity of lesions in the previous report), AA mouse model (induced by cell‐transferring or spontaneously affected) and immune status due to ageing (our AA‐affected mice were younger than 32 weeks old, but the mice in the previous report were 1 year old). IFN‐α production is activated by TLR7/9 signalling that requires the formation of a complex consisting of MyD88 (myeloid differentiation factor 88), TRAF3/6, IRAK1/4 and IRF7.^30^, ^31^, ^32^ We found that these adaptor molecules and transcriptional factors were also highly expressed in skin of AA‐affected mice, particularly in lesions of initial AA mice (Figure 1C–E). These data indicate that the IFN‐α signal pathway is locally activated in non‐lesional areas at the initial phase. A number of studies have suggested the relevance of TLRs to autoimmune diseases and to triggering IFN‐α production. HMGB1‐DNA immune complex activates TLR9 and induces pDC activation followed by IFN‐α secretion.^28^ HMGB1 is released from pDCs after stimulation and regulates IFN‐α production via RAGE in an autocrine way.^33^ HMGB1 is also shown to be released from damaged cells, based on *in vitro* study indicating that outer root sheath cells, which are non‐professional immune cells, secrete HMGB1 by poly(I:C)‐mediated TLR3 activation.^22^ In AA patients, HMGB1 levels in scalp and serum have been reported to be upregulated, compared with normal controls.^34^ HSP70 has also been reported to induce pDC activation and IFN‐α production.^29^ Moreover, the previous study for investigating the effect of heat treatment on AA development has suggested that induction of HSP70 may precipitate the development of AA in C3H/HeJ mice.^35^ We investigated whether these factors activate pDCs, and showed that mRNA expressions of HMGB1, RAGE and HSP70 were increased in lesions at the initial phase (Figure 1F–H). Serum level of HSP70 was also increased at the initial phase (Figure 1I). These results indicate that these factors might induce IFN‐α production in lesions at the initial phase of AA.

Although NLRP3 inflammasome has been reported to be associated with AA pathogenesis, little is known about its role in AA onset. Our data showed that IL‐1β level was increased in both serum and skin before and after AA onset (Figure 2A, E), whereas IFN‐α level was increased in lesions after AA onset (Figure 1B). Thus, the increase of IL‐1β preceded that of IFN‐α, which suggests that increase of IL‐1β production results in initiation of innate immune response around immune‐privileged HF. As well as IL‐1β production, IL‐18 production is also known to be regulated by NLRP3 inflammasome. Our data showed that IL‐18 production was increased in both lesions and non‐lesional areas of AA mice (Figure 2F). IL‐18 has been reported to induce Th1 and NK cell activation, IFN‐γ production,^36^ and type 1 chemokines.^37^ The blood level of *mmu*‐*miR*‐*150*‐*5p*, which downregulates IL‐18 expression, was lower before and after AA onset than in naive mice (Figure 2D), which is in concordance with the clinical report that the blood level of *hsa*‐*miR*‐*150*‐*5p* was decreased in AA patients.^38^ These data support the involvement of IL‐18 secretion in AA pathogenesis. The expressions of NLRP3 inflammasome components (NLRP3, ASC and caspase‐1) were highly increased in lesions at the initial phase and also upregulated in non‐lesional areas and lesions at the severe phase (Figure 2H–J). The increase of these factors in skin of AA‐affected mice is in agreement with clinical observations showing that NLRP3, ASC and caspase‐1 were significantly increased in the outer root sheath of HF in AA scalp skin.^22^ IFN‐γ is revealed to play a crucial role in the pathogenesis of AA and their levels in serum and skin tended to increase in mice injected with lymph node cells compared to naïve mice (Figure 2C, G). It is still unclear if IFN‐γ induces NLRP3 inflammasome or NLRP3 activation induces IFN‐γ secretion in this study. Whereas the level of IFN‐γ in skin was unchanged among pre‐AA, initial‐AA and severe‐AA, IL‐1β and IL‐18 protein levels in skin increased at initial and severe phases compared with preonset. Contribution of IL‐1β and IL‐18 to exacerbation of AA might be larger than that of IFN‐γ. Taken together, our data suggest that activation of NLRP3 inflammasome contributes to exacerbation as well as development of AA.

MCC950 is a potent, selective small molecule inhibitor of NLRP3, and its therapeutic effects on various autoimmune diseases have recently been anticipated.^39^ In the AA mouse model, MCC950 treatment prevented AA development and promoted hair growth (Figure 3A, B). The effect of MCC950 was observed just 7 days after first administration, and we investigated pathological characteristics on day 7. HE staining revealed that MCC950 treatment reduced infiltration of inflammatory cells such as neutrophils and lymphocytes (Figure 4A). The treatment also reduced expressions of type 1 chemokines, type 1 cytokine and cytotoxic marker, which were increased in AA‐affected mice, as well as NLRP3 inflammasome‐related factors (Figure 4B, C). Serum levels of IFN‐γ, TNF‐α and IL‐17A, which were increased in both AA patients and mice (data not shown), were decreased following MCC950 treatment (Figure 4D). IL‐1β is shown to inhibit hair elongation in HF culture.^40^ IL‐18 induces IFN‐γ production and, vice versa, IFN‐γ induces IL‐1β and IL‐18 production via NLRP3 inflammasome activation.^41^, ^42^ We speculate that MCC950 inhibits the negative loop of IFN‐γ and NLRP3 inflammasome and reduces Th1/Tc1 activation, consequently exerting a hair growth effect in AA mice. Our study sheds light on pharmacotherapy targeting the innate immune systems for AA, unlike the conventional pharmacological strategy, which targets T cells. Considering our finding that NLRP3 inflammasome activation contributes to not only AA exacerbation but also AA initiation, MCC950 might have not only have therapeutic effect but also preventive effect on AA relapse. Further studies will be needed to address whether NLRP3 inhibitor has a preventive effect against AA.

In conclusion, we reported that the IFN‐α‐producing pathway was activated in lesions at the initial phase, whereas the NLRP3 inflammasome pathway was activated before as well as after AA onset. Furthermore, administration of NLRP3 inflammasome inhibitor MCC950 induced hair regrowth and inhibited infiltration of inflammatory cells, expression of type 1 chemokines and cytokine in skin, and inflammatory cytokines in serum. The inhibitors of NLRP3 inflammasome may be candidates for novel therapeutic agents for AA.

## CONFLICT OF INTEREST

All authors are employees of Maruho Co., Ltd.

## AUTHOR CONTRIBUTIONS

KH designed the study, wrote the manuscript, performed the experiments and analysed the data. YY performed the experiments, reviewed and edited the manuscript. KS performed the experiments, analysed the data and reviewed the manuscript. SM and TM reviewed and edited the manuscript. All authors have read and approved the final manuscript.

## Data Availability

Research data are not shared.
